# Patterns of relative magnitudes of soil energy channels and their relationships with environmental factors in different ecosystems in Romania

**DOI:** 10.1038/srep17606

**Published:** 2015-12-01

**Authors:** Marcel Ciobanu, Iuliana Popovici, Jie Zhao, Ilie-Adrian Stoica

**Affiliations:** 1 Institute of Biological Research, Branch of the National Institute of Research and Development for Biological Sciences, 48 Republicii Street, 400015 Cluj-Napoca, Romania; 2 Key Laboratory of Agro-ecological Processes in Subtropical Region, Institute of Subtropical Agriculture, Chinese Academy of Sciences, Changsha 410125, PR China; 3 Huanjiang Observation and Research Station for Karst Ecosystems, Chinese Academy of Sciences, Huanjiang 547100, PR China

## Abstract

The percentage compositions of soil herbivorous, bacterivorous and fungivorous nematodes in forests, grasslands and scrubs in Romania was analysed. Percentages of nematode abundance, biomass and metabolic footprint methods were used to evaluate the patterns and relative size of herbivory, bacterial- and fungal-mediated channels in organic and mineral soil horizons. Patterns and magnitudes of herbivore, bacterivore and fungivore energy pathways differed for a given ecosystem type and soil depth according to the method used. The relevance of herbivore energy channel increased with soil depth due to higher contribution of root-feeders. Ectoparasites, sedentary parasites and epidermal cell and root hair feeders were the most important contributors to the total biomass and metabolic footprints of herbivores. Metabolic footprint method revealed the general dominance of bacterial-based energy channel in all five types of ecosystems. The influence of altitude and climatic factors on percentages of abundance, biomass and metabolic footprints of herbivores, bacterivores and fungivores decreased with soil depth, whereas the influence of humus content, cation-exchange capacity and base saturation increased. Vegetation, altitude, climate and soil physico-chemical characteristics are important factors that influenced the abundance, biomass and metabolic footprints of herbivores, bacterivores and fungivores.

Soils are essential components of all terrestrial ecosystems. They occupy a major role in the global interchange of matter and energy^1^, deliver key ecosystem services such as food production and contribute to climate mitigation^2^.

Soil biota is a key component of the soil food web and play fundamental role in ecosystem processes, like decomposition and humification^3^ and is considered to be the bottom-up driving force in ecosystems^4^,^5^. Since soil biota has a crucial role in ecosystem functioning^6^, certain groups of soil organisms are useful bioindicators for assessing soil functions and conditions^7^.

There are substantial evidences that species richness and diversity can enhance ecosystem functioning^8^,^9^, but since soil communities are highly diverse, their identification to species level requires a high level of expertise, is laborious, time consuming and costly^10^, making problematic the use of traditional taxonomical approach^11^. Therefore, using biodiversity surrogates as the higher taxon approach^7^,^12^,^13^, i.e. taxonomic identification to families/genera and focusing on the diversity of functional traits of species within a community seems a reasonable alternative, as it may improve the knowledge on the relationship between biodiversity and ecosystem functioning^8^,^9^,^14^,^15^.

Nematodes represent an important constituent of soil food webs^16^. They are a highly diverse group of soil invertebrates with vermiform body, mostly ranging between 0.2 and 2.5 mm^17^, cosmopolitan, abundant in soils, easy to collect and with fast response to changes in their environment^18^. Nematodes can be separated into eight trophic groups (plant feeders or herbivores, bacterial feeders or bacterivores, fungal feeders or fungivores, substrate ingesters, predators, unicellular eucaryote feeders, dispersal or infective stage of parasites and omnivores)^19^ as they show diversified feeding strategies and trophic specialization.

Since trophic diversity of soil nematodes covers all the three energy pathways which exist within the soil food web^20^, i.e. herbivory, bacterial- and fungal-mediated channels^21^, they are useful ecological tools to explore the functionality and complexity of soil food webs. Because root tissues and soil microorganisms (bacteria and fungi) represent primary energy sources for nematode communities, quantitative variation of these resources are thought to affect structural and trophic diversity of nematode fauna^22^. Apart from this, structural and functional diversity of plants (e.g. grass, forbs, legumes, trees etc.) and chemical content of plant roots are likely to induce changes in the composition of plant feeding nematodes^23^,^24^. Therefore, variability in the quality and quantity of resources in different ecosystems (e.g. grasslands, forests) can be reflected in the trophic structure of nematode communities^22^. Consequently, the relative abundance and/or biomass of plant feeding, bacterial feeding and fungal feeding nematodes can be assumed to represent their relative contribution to each of the three soil energy channels^25^.

Although much literature has been published on describing decomposition pathways in different ecosystem types by using the trophic diversity of nematode fauna^20^,^26^,^27^,^28^,^29^,^30^, the available information on the relative magnitude of soil energy channels, in particular herbivory pathway, is scarce^25^.

In this work we aim to use the percentage compositions of soil herbivorous, bacterivorous and fungivorous nematodes to analyse the patterns of relative magnitudes of soil energy channels at various soil depths in different ecosystem types in Romania in relation to environmental parameters. We hypothesized that: i) different ecosystem types are characterized by particular patterns of soil energy flows in relation to soil depth and quantity/quality of food resources available to nematode fauna; ii) patterns and relative magnitudes of soil energy channels at various soil depths are related with environmental variables, mostly with ecosystem type (i.e. vegetation), climatic factors and soil properties.

## Results

### The percentages of abundances of bacterivores, fungivores and herbivores in organic and mineral soil horizons

**Figure 1:**
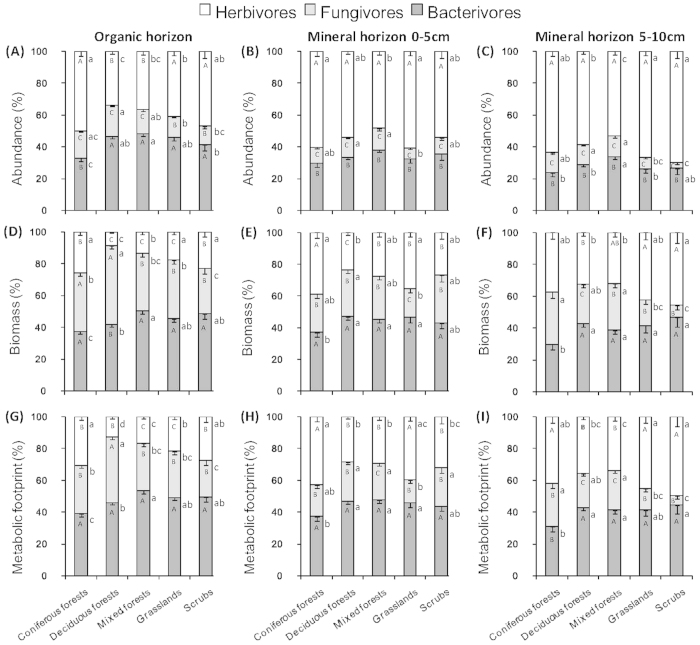
The percentages of abundance (**A–C**), biomass (**D–F**), and metabolic footprint (**G–I**) of the bacterivorous, fungivorous and herbivorous nematodes in organic horizon and 0–5 cm and 5–10 cm layers of mineral horizon of coniferous forest, deciduous forest, mixed forest, grassland, and scrub ecosystems in Romania. Bars represent means ± standard error. In each ecosystem, different uppercase letters indicate significant (P < 0.05) differences among the three nematode trophic groups. For each nematode trophic group, different lowercase letters indicate significant (P < 0.05) differences among ecosystem types. The total number of samples in each soil horizon and ecosystem type are given in Table 2.

The order of the percentage of nematode abundances was herbivore > bacterivore > fungivore in organic horizon of coniferous forests and scrubs and bacterivore > herbivore > fungivore in deciduous and mixed forests and grasslands (Fig. 1A). The contribution of bacterivores and herbivores was significantly higher than that of fungivores in organic horizon in both grasslands and scrubs. In addition, herbivores were significantly more abundant in coniferous forests than in deciduous forests, mixed forests and grasslands. The percentage of fungivores was significantly higher in deciduous forests than in grasslands and scrubs. The percentage of bacterivores was significantly higher in deciduous and mixed forests, grasslands and scrubs than in coniferous forests.

The order of the percentage of nematode abundances was herbivore > bacterivore > fungivore in mineral horizon in all five ecosystems (Fig. 1B and C). Herbivores had significant contribution to nematode communities in coniferous forests and grasslands, whereas contribution of fungivores was significantly higher in deciduous and mixed forests as compared to grasslands (Fig. 1B).

### The percentages of biomasses of bacterivores, fungivores and herbivores in organic and mineral soil horizons

The order of the percentage of nematode biomasses was bacterivore > fungivore > herbivore in organic horizon in coniferous forests, mixed forests, grasslands and scrubs and fungivore > bacterivore > herbivore in deciduous forests (Fig. 1D). The biomass of bacterivores and fungivores exceeded that of herbivores in coniferous forests. The percentages of the three trophic groups were significantly different from each other in mixed forests and grasslands.

The order of the percentage of nematode biomasses was herbivore > bacterivore > fungivore in the 0–5 cm layer of mineral horizon (Fig. 1E) in coniferous forests, and bacterivore > herbivore > fungivore in deciduous and mixed forests, grasslands and scrubs. The biomass of fungivores was higher in deciduous forests and scrubs, whereas that of bacterivores was greater in deciduous forests, mixed forests and grasslands.

The order of the percentages of nematode biomasses was herbivore > bacterivore > fungivore in the 5–10 cm layer of mineral horizon (Fig. 1F) in coniferous forests and grasslands and bacterivore > herbivore > fungivore in deciduous and mixed forests and scrubs. The biomass of fungivores was higher in coniferous and mixed forests, whereas that of bacterivores was greater in deciduous and mixed forests and scrubs.

### The percentages of metabolic footprints of bacterivores, fungivores and herbivores in organic and mineral soil horizons

The order of the percentage of nematode metabolic footprints was bacterivore > fungivore > herbivore in organic horizon of coniferous forests, mixed forests, grasslands and scrubs and fungivore > bacterivore > herbivore in deciduous forests (Fig. 1G.). Bacterivorous metabolic footprints were greater than herbivorous and fungivorous metabolic footprints in coniferous forests and scrubs. Bacterivorous and fungivorous metabolic footprints were significantly greater than that of herbivorous in deciduous forests.

The order of the percentage of nematode metabolic footprints was herbivore > bacterivore > fungivore in the 0–5 cm layer of mineral horizon in coniferous forests, and bacterivore > herbivore > fungivore in deciduous and mixed forests, grasslands and scrubs (Fig. 1H). Bacterivorous metabolic footprint was significantly greater than herbivorous and fungivorous metabolic footprints in deciduous forests and scrubs. Bacterivorous and herbivorous metabolic footprints were greater than fungivorous metabolic footprint in grasslands.

The order of the percentage of nematode metabolic footprints was herbivore > bacterivore > fungivore in the 5–10 cm layer of mineral horizon in coniferous forests and grasslands, and bacterivore > herbivore > fungivore in deciduous and mixed forests and scrubs (Fig. 1I). Herbivorous metabolic footprint was significantly higher than that of bacterivorous and fungivorous in coniferous forests. Bacterivorous and herbivorous metabolic footprints were greater than that of fungivorous in grasslands and scrubs.

### The percentages of abundance, biomass and metabolic footprints of different groups of plant feeders in organic and mineral soil horizons

**Figure 2:**
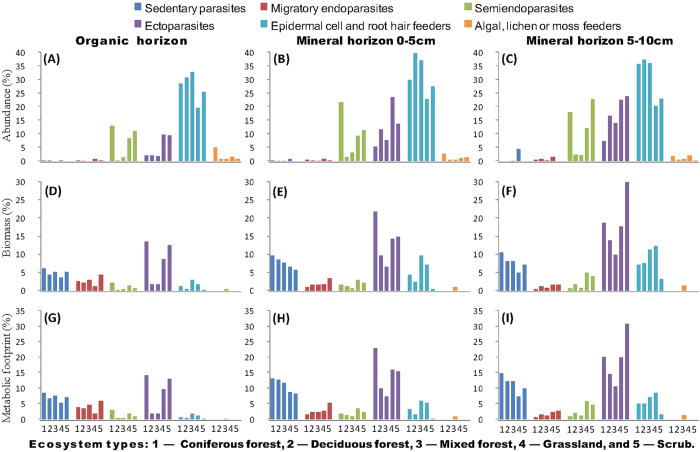
The percentages of abundance (**A–C**), biomass (**D–F**), and metabolic footprint (**G–I**) of different groups of plant feeders (i.e. sedentary parasites, migratory endoparasites, semiendoparasites, ectoparasites, epidermal cell and root hair feeders, and algal, lichen or moss feeders) in organic horizon, 0–5 cm and 5–10 cm layers of mineral horizon of coniferous forest, deciduous forest, mixed forest, grassland and scrub ecosystems in Romania. *Axonchium, Belondira, Dorylaimellus, Oxydirus* as well as juveniles belonging to Tylenchidae and Belondiridae were considered as plant feeders sensu lato and were excluded.

The percentages of abundance, biomass and metabolic footprints of plant feeding nematode groups in organic horizon, 0–5 cm and 5–10 cm layers in the five ecosystems were shown in Fig. 2.

The percentage of abundance of plant feeders (Fig. 2A–C) showed that epidermal cell and root feeders was by far the dominant group in organic soil horizon, being particularly favoured in forest and scrub soils, as compared to grassland soils. Semiendoparasites were more abundant in coniferous forests, scrubs and grasslands. Higher proportions of ectoparasites were found both in grassland and scrub soils. The abundance of these three groups increased, in general, with soil depth tested.

The percentages of biomass of plant feeders (Fig. 2D–F) showed higher proportions of ectoparasites in coniferous forests, scrubs and grasslands, followed by sedentary endoparasites, more numerous in coniferous forests, mixed forests and scrubs. The abundance of epidermal cell and root feeders increased, especially in mixed forests and grasslands. Scrub soils were characterized by the highest abundance of ectoparasites in the 5–10 cm layer of mineral soil horizon. Again, a general trend in increasing the percentages of biomass of ectoparasites, epidermal cell and root feeders and sedentary endoparasites with soil depth could be noted.

The percentages of metabolic footprints of plant feeders (Fig. 2G–I) revealed an almost similar pattern with the one showed for the percentages of biomass.

### Relationships between environmental variables and the percentages of abundance, biomass and metabolic footprints of bacterivores, fungivores and herbivores in organic and mineral soil horizons

**Figure 3:**
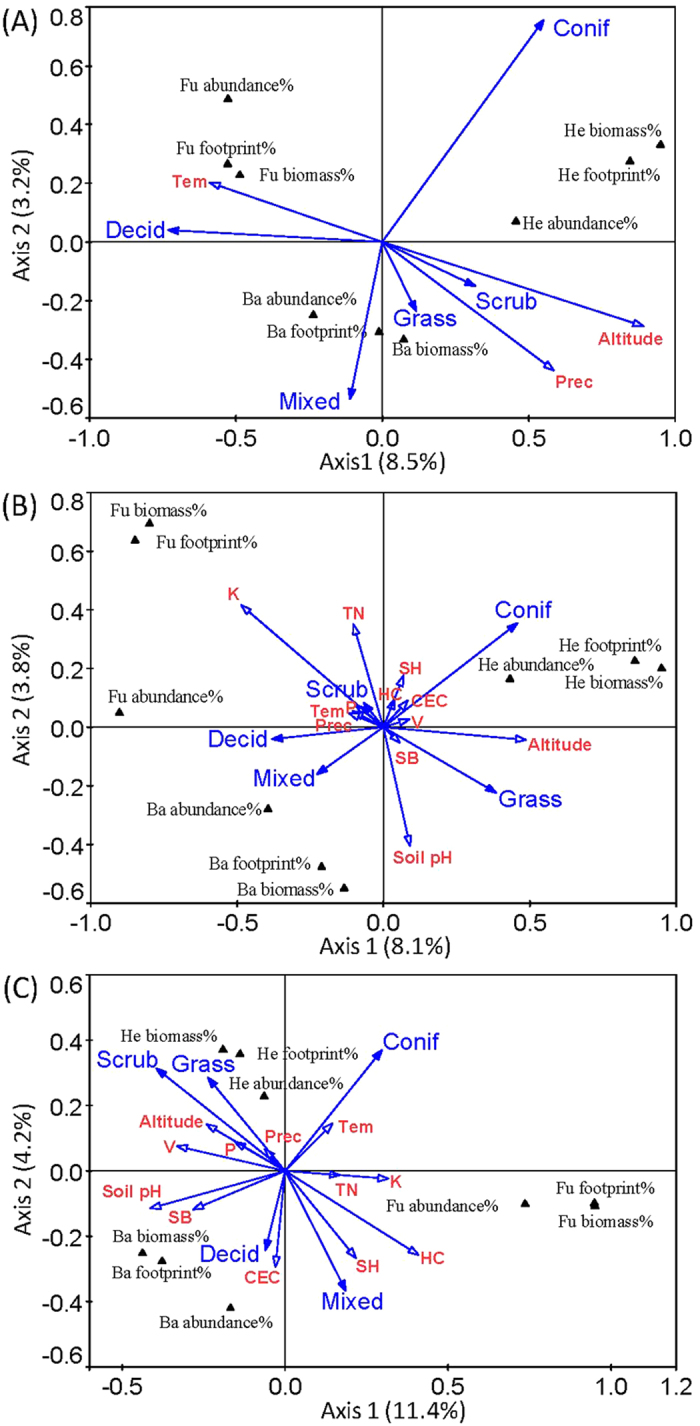
Bi-plots of canonical correspondence analysis (CCA) of the relationship between environmental variables (ecosystem types, soil physico-chemical properties, climatic variables, and altitude) and the percentages of abundance, biomass, and metabolic footprint of the bacterivorous, fungivorous and herbivorous nematodes in organic horizon (**A**) and 0–5 cm (**B**) and 5–10 cm (**C**) layers of mineral horizon. Ordination diagrams presenting species scores (triangles) and environmental factor scores (vectors). Solid vectors indicate ecosystem types, empty vectors indicate soil physico-chemical properties, climatic variables and altitude. Ba, bacterivore; Fu, fungivore; He, herbivore; Prec, annual average precipitation; Tem, annual average temperature; TN, total nitrogen; K, available potassium content; P, available phosphorus content; HC, humus content; CEC, cation-exchange capacity; SH, total hydrolytic acidity; SB, total exchangeable base; and V, base saturation. Environmental variables of organic horizon do not include soil physico-chemical properties. Monte Carlo permutation test results for the environmental variables were shown in Table 1.

Canonical correspondence analysis showed that the first two axes of the environmental variables explained 11.7%, 11.9%, and 15.6% of the variances of the percentages of abundance, biomass and metabolic footprints of bacterivores, fungivores and herbivores in organic horizon and in the 0–5 cm and 5–10 cm layers of mineral horizon, respectively (Fig. 3).

Monte Carlo permutation test results showed that different environmental variables were positively or negatively correlated with the percentages of abundance, biomass and metabolic footprints of bacterivores, fungivores and herbivores in organic and mineral soil horizons.

Altitude, coniferous forests, annual average precipitation and mixed forests significantly correlated with the percentages of abundance, biomass and metabolic footprints of bacterivores, fungivores and herbivores in organic horizon (P < 0.05) (Table 1 and Fig. 3A). Particularly, the altitude and annual average precipitation correlated negatively with fungivores (Fig. 3A). Mixed forests correlated positively with bacterivores and coniferous forests correlated negatively with the bacterivores (Fig. 3A).

Coniferous forests and grasslands significantly (P < 0.05) correlated with the percentages of abundance, biomass and metabolic footprints of bacterivores, fungivores and herbivores in the 0–5 cm layer of mineral horizon (Table 1 and Fig. 3B). Particularly, coniferous forests correlated positively with herbivores and negatively with the bacterivores (Fig. 3B). Grasslands correlated negatively with fungivores (Fig. 3B).

Coniferous forests, altitude, humus content, available potassium content, soil pH, cation-exchange capacity, and mixed forests significantly (P < 0.05) correlated with the percentages of abundance, biomass and metabolic footprints of bacterivores, fungivores and herbivores in the 5–10 cm layer of mineral horizon (Table 1 and Fig. 3C). Particularly, coniferous forests correlated negatively with bacterivores and soil pH correlated positively with bacterivores (Fig. 3C). Cation-exchange capacity correlated positively with bacterivores and negatively with herbivores (Fig. 3C). Mixed forests correlated negatively with herbivores (Fig. 3C). Humus content and available potassium content correlated positively with fungivores whereas altitude correlated negatively (Fig. 3C).

## Discussion

Nematode abundance, biomass and metabolic footprint methods generated different results of relative size of herbivore, fungivore and bacterivore energy channels for a given ecosystem type and soil depth.

This outcome is different from the one reported by Zhao and Neher^25^,who described similar patterns of soil energy channel in grasslands, croplands and forests when nematode abundance and biomass methods were used.

There was a clear distinction between the relative magnitudes of herbivore, bacterivore and fungivore energy channels when comparing the three methods. When nematode abundance method was used, the relative magnitudes of herbivore energy channel appeared much greater, but much lower for bacterivore and fungivore energy channels as compared to the biomass and metabolic footprint methods. As a result, nematode abundance method overestimated the herbivory mediated channel and underestimated the bacterial- and fungal-mediated pathways. Although epidermal cell and root hair feeders were the most abundant plant feeders (Fig. 2A,B), their biomasses (Fig. 2D–F) and metabolic footprints (Fig. 2G–I) were very low.

Interestingly, biomass and metabolic footprint methods generated similar patterns among the five ecosystem types for a given energy channel and soil depth. This result is attributed to the fact that biomass is closely related to the carbon utilization and the magnitudes of soil energy channels. Therefore, biomass and metabolic footprint methods may be more appropriate for evaluating soil energy channels as compared to nematode abundance method because they are related to matter and energy flows in the ecosystems. On the contrary, nematode abundance method does not take into account body size and carbon utilization by nematodes with different functional characteristics^31^ and using this method in evaluating soil energy channels may be biased due to large variation in nematode body sizes or biomasses.

The factors that influence the soil energy channels are complex (Supplementary information). Vegetation identity had been shown to have strong impacts on soil nematode fauna^23^,^31^,^32^ and was thought to have major role in controlling energy channels in soil systems^24^. Consistently, ecosystem type—characterized by vegetation composition—affected the energy channels in the present study (Supplementary information). Particularly ecosystem type could directly and/or indirectly affect the energy channels via differentiation in soil resource (Supplementary information, Fig. S1B). In forests, basal resources such as leaf litter and root-derived products are considered to be important in structuring soil food webs^24^.

Different quality and quantity of basal resources related to tree composition and understory vegetation in forest ecosystems (coniferous, deciduous and mixed) resulted in different patterns and magnitudes of herbivore, fungal and bacterivore energy channels, in line with the first hypothesis. However, remarkably, there are no striking differences regarding the patterns and relative size of the three energy channels between forest types and between forests, grasslands and scrubs. This result suggests that grouping sites according to plant associations, which are strongly related to soil characteristics and parent rock, may be more appropriate when evaluating soil energy channels of different ecosystems.

The importance of the herbivore energy channel increased in order organic layer < mineral 0–5 cm layer < mineral 5–10 cm layer, the shift being more evident when comparing organic soil horizon to mineral 0–5 cm layer and less obvious among the two layers (0–5 cm and 5–10 cm) of mineral soil horizon. The increasing importance of herbivore energy channel in mineral soil horizon was in relation to the higher contribution of root-feeding nematodes, more abundant in deeper soil layers. The importance and relevance of herbivore energy channel in grassland and forest ecosystems has previously been addressed by Zhao and Neher^25^, suggesting that it is responsible for a considerable part of the below-ground energy flow. The results shown here are therefore in line with the previous findings and stress the increasing importance of herbivorous-mediated channel in relation to soil depth.

Distribution of different groups of plant feeders associated with soil horizons is thought to reflect the distribution of plant roots at various soil depths^33^,^34^. Vertical migration of certain plant feeders to more stable and uniform conditions in deeper soil layers is believed to be a strategy to avoid fluctuations in soil moisture and temperature in upper soil horizons despite a reduced availability of feeding sites^35^.

Evaluated by the metabolic footprint method, the five ecosystems were generally dominated by the bacterial-based energy channel, suggesting bottom-up regulation (Wang, pers. comm.), except the coniferous forests in the 5–10 cm layer of mineral horizon, where herbivorous-based energy channel prevailed. In addition, the relative sizes of the fungal-based energy channel in organic horizon of deciduous forests, the herbivorous-based energy channels in the 0–5 cm layer of mineral horizon of coniferous forests and grasslands, and the herbivorous-based energy channels in the 5–10 cm layer of mineral horizon of grasslands and scrubs were similar to the relative sizes of their corresponding bacterial-based energy channels of an ecosystem. In general, the size of the fungal-based energy channel in organic horizon was relatively greater than that of the herbivorous-based energy channel, except the coniferous forests and scrubs. This pattern was reversed in the 0–5 cm and 5–10 cm layers of mineral horizon, where herbivorous-based energy channel increased in importance. The general dominance of bacterial-based energy channel in all five types of ecosystems surveyed is congruent with previous findings^25^,^27^,^36^,^37^,^38^,^39^,^40^,^41^, indicating resource-rich substrates.

Unravelling patterns of interactions between soil energy channels and environmental factors is both challenging and captivating because offer insights on how soil food webs are driven and function. As noted above, various factors influenced soil energy channels, although resource quantity and quality may be the main controlling factors for a given ecosystem type^23^,^24^,^31^,^32^ at small scale. However, at regional scale different environmental factors interacted, resulting more complex effects on energy pathways (Supplementary information). Specifically, not only soil resource but also climate, ecosystem type and soil environment showed significant influences on soil energy channels in forests, scrubs and grasslands. In organic horizon, the proportion of fungivores declined with altitude and precipitation, in line with previous findings^42^, probably as result of resource limitation due to climatic influence. The negative influence of altitude on fungivores maintained as well in the 5–10 cm layer of mineral soil horizon. Bacterivores were favoured in mixed forests, but responded negatively to acidic substrate of coniferous forests, more favourable to plant-feeding nematodes in the 0–5 cm layer of mineral soil horizon. The negative correlation pattern of bacterivores with coniferous forests was found in both organic and mineral soil horizons, suggesting that soil pH resulting from high phenol content of coniferous litter^43^,^44^,^45^ is a strong regulator of bacterial feeding nematode populations in this type of ecosystem. Soil pH has previously been found to be an important limiting factor for survival of bacterial feeding nematodes^46^. Although higher amounts of organic matter were reported in coniferous stands as compared to deciduous and mixed ones, the concentration of microorganisms was much higher in deciduous litter^47^. This point towards a direct link between the densities of bacteria and bacterivores in soils of coniferous forests.

As expected, the influence of altitude and climatic factors on percentages of abundance, biomass and metabolic footprints of herbivores, fungivores and bacterivores decreased with soil depth, whereas the influence of certain soil physico-chemical parameters (e.g. humus content, cation-exchange capacity, base saturation) increased.

The results bring new information on how different biotic and abiotic factors correlated with percentages of abundance, biomass and metabolic footprints of herbivores, fungivores and bacterivores at various soil depths. They reveal that vegetation, altitude, climate and soil physico-chemical characteristics are important factors that influence the abundance, biomass and metabolic footprints of herbivores, fungivores and bacterivores in forests, scrubs and grasslands, in line with the second hypothesis.

## Concluding remarks

The analysis of trophic diversity of nematode communities in five ecosystem types (coniferous forests, deciduous forests, mixed forests, grasslands and scrubs) in Romania revealed different patterns and magnitudes of bacterivore, fungivore and herbivore energy channels in organic and mineral soil horizons.

Different relative sizes of herbivore, fungivore and bacterivore energy pathways for a given ecosystem type and soil depth were assessed when percentages of nematode abundance, biomass and metabolic footprints methods were used. Biomass and metabolic footprint methods generated similar patterns among the five ecosystem types for a given energy channel and soil depth. These two methods are considered to be more appropriate for assessing soil energy channels as they are more connected to C and energy flows in soil systems.

The role and relevance of herbivore energy channel increased in all ecosystem types with soil depth due to higher contribution of root-feeders in mineral soil horizon as compared to organic soil horizon. Ectoparasites, sedentary parasites and epidermal cell and root hair feeders were the most important contributors to the total biomass and metabolic footprints of herbivores.

Metabolic footprint and biomass methods were more appropriate in evaluating the relative magnitudes of soil energy channels and both of them revealed the general dominance of bacterial-based energy channel in all five types of ecosystems.

The influence of altitude and climatic factors on percentages of abundance, biomass and metabolic footprints of herbivores, fungivores and bacterivores decreased with soil depth, whereas the influence of humus content, cation-exchange capacity and base saturation increased.

The results showed that vegetation, altitude, climate and soil physico-chemical characteristics are important factors that influence the abundance, biomass and metabolic footprints of bacterivores, fungivores and herbivores in forests, scrubs and grasslands.

Resource quantity and quality may be the main driving factors of soil energy channels in ecosystems at small scale, but multiple interactions between various environmental factors at regional scale result in more complex effects on energy pathways.

## Materials and Methods

### Site description

A total of 135 sites distributed in the Romanian Carpathians (128 sites) and the Transylvanian Plateau (7 sites) were investigated. They included representative coniferous (n = 27), deciduous (n = 43) and mixed (n = 26) forests, grasslands (n = 31), scrubs (n = 7) and also a European black pine (*Pinus nigra* Arnold) plantation. Coniferous forests consisted mostly of Norway spruce (*Picea abies* L.). Most of the deciduous forests included beech (*Fagus sylvatica* L.), alone or in association with hornbeam (*Carpinus betulus* L.); other species were sessile oak (*Quercus petraea* (Matt.) Leibl) and sweet chestnut (*Castanea sativa* Mill.). Mixed forests were mostly composed of Norvegian spruce (*Picea abies* L.), beech (*Fagus sylvatica* L.), and occasionally silver fir (*Abies alba* Mill.). Mountain dwarf pine (*Pinus mugo* Turra.) was present in all scrubs. The sites were situated along an altitudinal gradient from 180 to 2,350 m a.s.l., those located higher in the Carpathians were generally less affected by human activities, some of them being located in pristine natural protected areas. Sampling was carried out between 1974 and 1999, during an extensive survey focused on Romanian biodiversity.

### Sampling, extraction, identification

Three to seven 25 m^2^ plots were selected at random from a surface of about 500 m^2^, which was considered to be representative for the ecosystem type. Roughly, the distances between plots were at least 10 m and therefore sample replicates were in fact pseudoreplicates. In each plot, one sample comprising 10 cores (cores of 25 cm^2^ in surface) was collected from the organic horizons (O and AO respectively, see explanation below) and A layer (but sometimes also from B layer, if present) of the mineral soil horizon (0–5 cm and 5–10 cm; cores of 3.8 cm^2^ in surface), separately. This resulted in a total of 597 samples including organic horizons only, 378 samples containing exclusively the top 5 cm of the A layer of mineral horizon and 291 samples including solely the 5–10 cm of the A (and sometimes B) layer of mineral horizon, respectively (Table 2). According to the Romanian System of Soil Classification^48^, the organic horizon (O) included litter, fermentation and humification layers (which characterized most forests), while the sod (or turf) horizon (AO) was the shallow surface soil horizon with high proportion of matted roots in grassland soils.

Nematodes in these samples were extracted using the centrifugation method^49^, after which they were counted and fixed with 4% formaldehyde solution heated at 65°C. Temporary mass-slides were prepared from each sample and used for examination. At least 150 individuals were randomly identified at the microscope at 400 × magnification and were used to estimate the relative abundance. Adults were identified to genus level whereas juveniles were generally assigned to families. The classification system used was according to Andrássy^50^, Siddiqi^51^ and Bongers^52^. The contribution of trophic groups was established according to Yeates *et al.*^19^

A separate set of samples from the A layer of mineral horizon was taken for assessing soil physico-chemical properties, as this horizon was considered less exposed to climatic factors and with more stable and uniform conditions. Standard methods were used for the determination of soil pH (in water), available phosphorus (P_2_O_5_) (ppm) and potassium (K_2_O; ppm), humus content (%), total nitrogen content (%), total exchangeable bases (SB; (meq 100 g^−1^ soil), total hydrolytic acidity (SH; meq 100 g^−1^ soil), cation-exchange capacity (CEC; %), and base saturation (V; %)^53^,^54^.

Data on annual average temperature and precipitation was compiled from the CARPATCLIM—Climate of the Carpathian Region online database (CARPATCLIM Database, 2013; www.carpatclim-eu.org) for each sampling site, based on its geographical coordinates and using a running mean (e.g. sampling year −11 up to sampling year −1), as sampling was done in several field campaigns from 1974–1999.

### Data analysis

Three trophic groups of nematodes (plant feeding, bacterial feeding and fungal feeding) were taken into account to evaluate soil energy channels^25^. Omnivore and predatory nematodes were excluded because they are located in higher trophic levels in soil food webs than bacterivores, fungivores and herbivores and are not the most relevant or direct groups to assess the relative magnitudes of the herbivory, bacterial- and fungal-mediated channels^5^,^25^. Percentages of abundance, biomass and metabolic footprint were calculated for each of these trophic groups in each sample. Mean nematode biomass of a genus was assigned as mean biomass of the respective taxonomic family^55^. When mean biomass of the respective taxonomic family for a given genus was not provided in Ferris^55^, biomass of the genus given in Zhao *et al.*^56^,^57^ was used. Calculation of metabolic footprint for each trophic group was as follows:


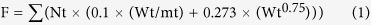


where Nt is abundance of the t taxa in a sample, Wt is the biomass of the t taxa, and mt is the cp-value of the t taxa. Percentages of the abundance, biomass and metabolic footprint of plant feeding, bacterial feeding and fungal feeding nematodes in the five ecosystem types, at different soil depths were analyzed separately by one-way ANOVA. Briefly, the differences of a given energy channel among ecosystems at each soil depth (27 variables, 3 trophic groups × 3 soil depths × 3 methods) and the differences among the three energy channels for a given ecosystem at a given soil depth (45 variables, 5 ecosystems × 3 soil depths × 3 methods) were analyzed. The percentage data were firstly arcsine transformed to meet assumptions of normality and homogeneity of variance. Alternatively, the data were natural log, square root, or rank transformed to meet the assumptions above. Canonical correspondence analysis (CCA)^58^ was performed using CANOCO 4.5 software (Ithaca, NY, USA) to determine the relationships between soil energy pathways and environmental variables, and between soil energy pathways and ecosystem types, with environmental variables as co-variables. Statistical significance was determined at P < 0.05. ANOVAs were performed using SPSS software version 16.0 (SPSS Inc., Chicago, IL, USA). LSD was used to test differences among treatment means; and Tamhane’s T2 was used to test differences among treatments when variances of transformed data were unequal. Forward selection and test of significance for the first axis were based on Monte Carlo permutation test (n = 499).

## Additional Information

**How to cite this article**: Ciobanu, M. *et al.* Patterns of relative magnitudes of soil energy channels and their relationships with environmental factors in different ecosystems in Romania. *Sci. Rep.* **5**, 17606; doi: 10.1038/srep17606 (2015).

## Supplementary Material

Supplementary Information

## Figures and Tables

**Table 1:** Monte Carlo permutation test results of canonical correspondence analysis (CCA) of the relationship between environmental variables (ecosystem types, soil physico-chemical properties, climatic variables and altitude) and the percentages of abundance, biomass, and metabolic footprint of bacterivorous, fungivorous and herbivorous nematodes in organic horizon and 0–5 cm and 5–10 cm layers of mineral horizon.

Environmental variables	Organic horizon		Mineral 0–5 cm		Mineral 5–10 cm	
	*F*	*P*	*F*	*P*	*F*	*P*
Coniferous forest (Conif)	22.34	0.002	7.60	0.002	7.33	0.002
Deciduous forest (Decid)	2.09	0.124	1.09	0.350	1.55	0.176
Mixed forest (Mixed)	3.00	0.044	–	–	3.02	0.048
Grassland (Grass)	–	–	5.51	0.002	–	–
Scrub	–	–	1.88	0.146	–	–
Altitude	45.03	0.002	2.83	0.052	6.70	0.004
Annual average precipitation	4.02	0.010	2.47	0.076	1.37	0.234
Annual average temperature	–	–	2.34	0.072	0.92	0.406
Humus content (HC)	/	/	1.19	0.316	6.55	0.002
Total nitrogen (TN)	/	/	1.93	0.134	1.38	0.266
Soil pH	/	/	2.06	0.116	5.05	0.008
Available phosphorus content (P)	/	/	0.55	0.586	2.29	0.068
Available potassium content (K)	/	/	0.75	0.492	6.54	0.002
Cation-exchange capacity (CEC)	/	/	4.55	0.006	4.43	0.008
Total hydrolytic acidity (SH)	/	/	1.83	0.160	0.64	0.538
Total exchangeable base (SB)	/	/	4.36	0.012	0.58	0.606
Base saturation (V)	/	/	2.47	0.068	2.49	0.078

Abbreviation in parentheses corresponding to Fig. 3; – indicates that further addition of the variable via forward selection did not improve model fitting and the Monte Carlo permutation test did not perform for the respective variable; /indicates that soil physico-chemical properties were only assessed for mineral horizon.

**Table 2:** Sampling locations and number of samples in organic horizon, 0–5 cm and 5–10 cm layers of mineral horizon of different ecosystems in Romania (the coniferous forest at Cluj consisted in a *Pinus nigra* Arnold plantation).

Ecosystem type	Number of samples			Sampling location (except Cluj, all siteslocated in mountains)
	Organic horizon	Mineral 0–5 cm	Mineral 5–10 cm	
Coniferous forests	150	69	43	Aninei, Apuseni, Căliman, Ceahlău, Ciucaş, Cluj, Giumalău-Rarău, Gurghiu, Harghita, Hăşmaş, Maramureşului, Parâng, Retezat, Rodna and Vrancei
Deciduous forests	167	144	129	Aninei, Apuseni, Bârgăului, Căliman, Ceahlău, Ciucaş, Cluj, Făgăraş, Harghita, Maramureşului, Parâng, Retezat, Rodnei, Transylvanian Plateau and Vrancei
Mixed forests	117	89	78	Aninei, Apuseni, Bârgăului, Bistriţei, Buzăului, Căliman, Făgăraş, Gurghiu, Maramureşului, Parâng, Retezat, Rodnei and Vrancei
Grasslands	114	44	24	Apuseni, Bârgăului, Căliman, Ciucaş, Făgăraş, Giumalău-Rarău, Gurghiu, Harghita, Hăşmaş, Parâng, Retezat and Rodnei
Scrubs	49	32	17	Retezat and Rodnei
Total	597	378	291	

## References

[b1] Filip Z. International approach to assessing soil quality by ecologically-related biological parameters. Agric Ecosyst Environ. 88, 169–174 (2002).

[b2] De Vries F. T. *et al.* Soil food web properties explain ecosystem services across European land use systems. Proc Natl Acad Sci USA 110, 14296–14301 (2013). 23940339 10.1073/pnas.1305198110 PMC3761618

[b3] Brussaard L., Pulleman M. M., Ouédraogo É., Mando A. & Six J. Soil fauna and soil function in the fabric of the food web. Pedobiologia. 50, 447–462 (2007).

[b4] Hunt H. & Wall D. Modelling the effects of loss of soil biodiversity on ecosystem function. Global Change Biol. 8, 33–50 (2002).

[b5] Scheu S. & Setälä H. Multitrophic interactions in decomposer food-webs. In: Tscharntke T., Hawkins B. A., editors. Multitrophic level interactions pp. 223–264 (Cambridge University Press, Cambridge 2002).

[b6] Copley J. Ecology goes underground. Nature. 406, 452–454 (2000). 10952284 10.1038/35020131

[b7] Bhusal D. R., Kallimanis A. S., Tsiafouli M. A. & Sgardelis S. P. Higher taxa vs. functional guilds vs. trophic groups as indicators of soil nematode diversity and community structure. Ecol Indic. 41, 25–29 (2014).

[b8] Cardinale B. J. *et al.* Biodiversity loss and its impact on humanity. Nature. 486, 59–67 (2012). 22678280 10.1038/nature11148

[b9] Hooper D. U. *et al.* Effects of biodiversity on ecosystem functioning: a consensus of current knowledge. Ecol Monogr. 75, 3–35 (2005).

[b10] Lawton J. H. *et al.* Biodiversity inventories, indicator taxa and effects of habitat modification in tropical forest. Nature. 391, 72–76 (1998).

[b11] Jeffery S. *et al.* European atlas of soil biodiversity. (European Commission, 2010).

[b12] Balmford A., Green M. & Murray M. Using higher-taxon richness as a surrogate for species richness: I. Regional tests. P Roy Soc B Biol Sci. 263, 1267–1274 (1996).

[b13] Gaston K. J. & Williams P. H. Mapping the world’s species-the higher taxon approach. Biodiv Lett. 1, 2–8 10.2307/2999642 (1993).

[b14] Cadotte M. W., Carscadden K. & Mirotchnick N. Beyond species: functional diversity and the maintenance of ecological processes and services. J Appl Ecol. 48, 1079–1087 (2011).

[b15] Díaz S. & Cabido M. Vive la difference: plant functional diversity matters to ecosystem processes. Trends Ecol Evol. 16, 646–655 (2001).

[b16] Bonkowski M. Protozoa and plant growth: the microbial loop in soil revisited. New Phytol. 162, 617–631 (2004). 10.1111/j.1469-8137.2004.01066.x 33873756

[b17] Mulder C. & Vonk J. A. Nematode traits and environmental constraints in 200 soil systems: scaling within the 60–6000 μm body size range: Ecological Archives E092-171. Ecology. 92, 2004–2004 (2011).

[b18] Bongers T. & Ferris H. Nematode community structure as a bioindicator in environmental monitoring. Trends Ecol Evol. 14, 224–228 (1999). 10354624 10.1016/s0169-5347(98)01583-3

[b19] Yeates G., Bongers T., De Goede R., Freckman D.& Georgieva S. Feeding habits in soil nematode families and genera—an outline for soil ecologists. J Nematol. 25, 315 (1993). 19279775 PMC2619405

[b20] Ferris H., Bongers T. & De Goede R. A framework for soil food web diagnostics: extension of the nematode faunal analysis concept. Appl Soil Ecol. 18, 13–29 (2001).

[b21] Sylvain Z. A. *et al.* Soil animal responses to moisture availability are largely scale, not ecosystem dependent: insight from a cross‐site study. Global Change Biol. 20, 2631–2643 (2014). 10.1111/gcb.12522 24399762

[b22] Biederman L. A. & Boutton T. W. Biodiversity and trophic structure of soil nematode communities are altered following woody plant invasion of grassland. Soil Biol Biochem. 41, 1943–1950 (2009).

[b23] De Deyn G. B., Raaijmakers C. E., Van Ruijven J., Berendse F. & Van Der Putten W. H. Plant species identity and diversity effects on different trophic levels of nematodes in the soil food web. Oikos. 106, 576–586 (2004).

[b24] Cesarz S. *et al.* Tree species diversity versus tree species identity: driving forces in structuring forest food webs as indicated by soil nematodes. Soil Biol Biochem. 62, 36–45 (2013).

[b25] Zhao J. & Neher D. A. Soil energy pathways of different ecosystems using nematode trophic group analysis: a meta analysis. Nematology. 16, 379–385 (2014).

[b26] Freckman D. W. & Ettema C. H. Assessing nematode communities in agroecosystems of varying human intervention. Agric Ecosyst Environ. 45, 239–261 (1993).

[b27] Ruess L. Nematode soil faunal analysis of decomposition pathways in different ecosystems. Nematology. 5, 179–181 (2003).

[b28] Yeates G. W. Nematodes as soil indicators: functional and biodiversity aspects. Bio Fert Soils. 37, 199–210 (2003).

[b29] Popovici I. Abundance, biomass and energetic parameters of soil nematode populations in a spruce forest (Bihor Mountains) [in Romanian]. Actuality and perspective in biology Structure and functions in terrestrial and aquatic ecosystems pp. 71–78 (Centre of Biological Research, Cluj-Napoca 1985).

[b30] Popovici I. Structure and dynamics of nematode communities (Nematoda) [in Romanian]. The Retezat National Park-Ecological Studies pp. 200–214 (Editura West Side, 1993).

[b31] Sohlenius B., Boström S. & Viketoft M. Effects of plant species and plant diversity on soil nematodes–a field experiment on grassland run for seven years. Nematology. 13, 115–131 (2011).

[b32] Viketoft M. *et al.* Long-term effects of plant diversity and composition on soil nematode communities in model grasslands. Ecology. 90, 90–99 (2009). 19294916 10.1890/08-0382.1

[b33] Assheuer T. & Roessner J. Is the abundance of plant-parasitic nematodes correlated with the root biomass of hosts ? Med Fac Landbouwwetensch Rijksuniv Gent. 58, 719–728 (1993).

[b34] Yeates G. How plants affect nematodes. Adv Ecol Res. 17, 61–113 (1987).

[b35] Verschoor B. C., De Goede R. G., De Hoop J.-W. & De Vries F. W. Seasonal dynamics and vertical distribution of plant-feeding nematode communities in grasslands. Pedobiologia. 45, 213–233 (2001).

[b36] Háněl L. Recovery of soil nematode populations from cropping stress by natural secondary succession to meadow land. Appl Soil Ecol. 22, 255–270 (2003).

[b37] Popovici I. & Ciobanu M. Diversity and distribution of nematode communities in grasslands from Romania in relation to vegetation and soil characteristics. Appl Soil Ecol. 14, 27–36 (2000).

[b38] Sohlenius B. Abundance, biomass and contribution to energy flow by soil nematodes in terrestrial ecosystems. Oikos. 34, 186–194 10.2307/3544181 (1980).

[b39] Wasilewska L. Structure and function of soil nematode communities in natural ecosystems and agrocenoses. Pol Ecol Stud. 27, 97–146 (1979).

[b40] Yeates G. & Bird A. Some observations on the influence of agricultural practices on the nematode faunae of some South Australian soils. Fund Appl Nematol. 17, 133–145 (1994).

[b41] Zhao J. & Neher D. A. Soil nematode genera that predict specific types of disturbance. Appl Soil Ecol. 64, 135–141 (2013).

[b42] Yeates G. & Bongers T. Nematode diversity in agroecosystems. Agric Ecosyst Environ. 74, 113–135 (1999).

[b43] Gallet C. & Lebreton P. Evolution of phenolic patterns in plants and associated litters and humus of a mountain forest ecosystem. Soil Biol Biochem. 27, 157–165 (1995).

[b44] Lorenz K., Preston C. M., Raspe S., Morrison I. K. & Feger K. H. Litter decomposition and humus characteristics in Canadian and German spruce ecosystems: information from tannin analysis and 13 C CPMAS NMR. Soil Biol Biochem. 32, 779–792 (2000).

[b45] Ulrich B. Nutrient and acid-base budget of central European forest ecosystems. In: Godbold D. L., Hüttermann A., editors. Effects of acid rain on forest processes pp. 1–50 (Wiley, New York 1994).

[b46] Liang W. *et al.* Vertical distribution of bacterivorous nematodes under different land uses. J Nematol. 37, 254 (2005). 19262869 PMC2620973

[b47] Scheu S. *et al.* The soil fauna community in pure and mixed stands of beech and spruce of different age: trophic structure and structuring forces. Oikos. 101, 225–238 (2003).

[b48] Conea A., Florea N.& Puiu S. Romanian System of Soil Classification [in Romanian]. (Institutul de Cercetări Pedologice şi Agronomice, 1980).

[b49] De Grisse A. T. Redescription ou modifications de quelques techniques utilises dans l'étude des nematodes phytoparasitaires. Meded Fak Landbouww Gent. 34, 351–369 (1969).

[b50] Andrássy I. Klasse Nematoda. Bestimmungsbücher zur bodenfauna Europas. Lieferung 9. (Akademie Verlag, Berlin 1984).

[b51] Siddiqi M. R. Tylenchida: parasites of plants and insects. (CABI, 2000).

[b52] Bongers T. De Nematoden Van Nederland . (Natuurhistorische Bibliotheek van de KNNV, nr. 46, Pirola, Schoorl 1988).

[b53] Carter M. R. Soil sampling and methods of analysis. (CRC Press, 1993).

[b54] Kalra Y. P.& Maynard D. G. Methods manual for forest soil and plant analysis. (1991).

[b55] Ferris H. Form and function: metabolic footprints of nematodes in the soil food web. Eur J Soil Biol. 46, 97–104 (2010).

[b56] Zhao J. *et al.* Size spectra of soil nematode assemblages under different land use types. Soil Biol Biochem. 85, 130–136 (2015).

[b57] Zhao J. *et al.* Using the biomasses of soil nematode taxa as weighting factors for assessing soil food web conditions. Ecol Indic. 60, 310–316 (2016).

[b58] Lepš J.& Šmilauer P. Multivariate analysis of ecological data using CANOCO. pp. xii+269 pp. (Cambridge University Press, New York 2003).

