# Supplementary material for: Patterns of relative magnitudes of soil energy channels and their relationships with environmental factors in different ecosystems in Romania
Source: Sci Rep. 2015 Dec 1;5:17606. doi: 10.1038/srep17606 (PMC4664958; doi:10.1038/srep17606)
Supplement: Supplementary Information [file srep17606-s1.pdf]

SUPPLEMENTARY INFORMATION

Patterns of relative magnitudes of soil energy channels and their relationships with environmental factors in different ecosystems in Romania

Marcel Ciobanu, Iuliana Popovici, Jie Zhao, Ilie-Adrian Stoica

Supplementary Figure S1 Final model results of structural equation modelling (SEM) analysis for the influence factors on bacterivorous, fungivorous and herbivorous nematodes in organic horizon (A) and 0-5 cm (B) and 5-10 cm (C) layers of the mineral horizon. Square boxes denote variables included in the models. Circles (e1–e6) indicate error terms, and double-headed arrows indicate significant correlations between the error terms. Results of model fitting for organic horizon: χ^2=631.454, P=0.000, d.f.=1; results of model fitting for 0-5 cm layer of mineral horizon: χ^2=0.117, P=0.732, d.f.=1; results of model fitting for 5-10 cm layer of mineral horizon: χ^2=0.589, P=0.443, d.f.=1. Solid arrows denote significant (P<0.05) directions and effects; dashed arrows represent not significant (P>0.05) directions and effects. Values associated with solid arrows represent standardized path coefficients. Data fitted to the models using the maximum likelihood estimation method using IBM SPSS Amos 21.0.0 (Amos Development Corporation; Meadville, PA, USA) to parameterize the model. The chi-square goodness-of-fit statistic and its associated P value were used to judge the model fit to the data. A large P value (>0.05) associated with the chi-square value (χ^2) indicates that the covariance structure of the data does not differ significantly from the expected, based on the model[58]. Prior to SEM procedure, we reduced the number of variables for climate, ecosystem type, resource, soil environment, bacterivores, fungivores and herbivores separately using principal component analysis (PCA). For each group, the first principal component (PC1) was used in the subsequent SEM analysis (% of variance for each variable explained by PC1 are given in parentheses). Climate variables include annual average precipitation, annual average temperature, and altitude; resource variables include humus content, total nitrogen, available phosphorus content, and available potassium content; soil environment variables include soil pH, cation-exchange capacity, total exchangeable base, total hydrolytic acidity, and base saturation; bacterivore, fungivore and herbivore variables include the percentages of nematode abundance, biomass, and metabolic footprint of the respective trophic group.

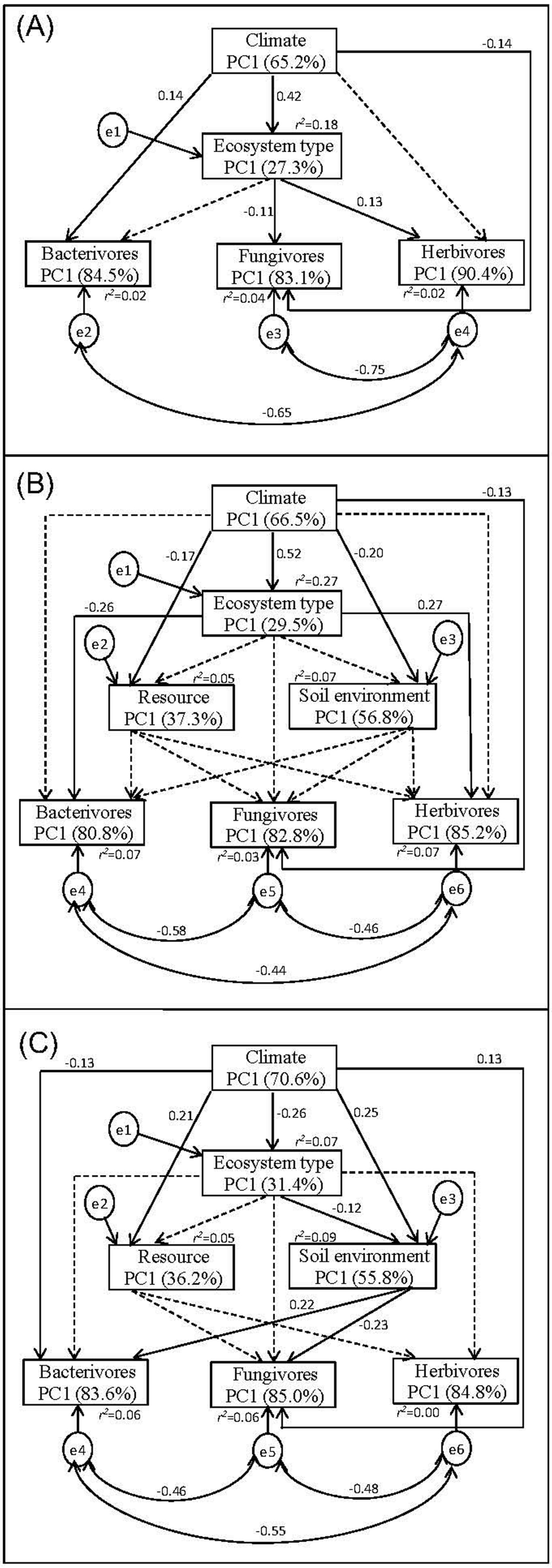
(A)
Climate
PC1 (65.2%)
-0.14
0.14
0.42
e1
r^2=0.18
Ecosystem type
PC1 (27.3%)
-0.11
0.13
Bacterivores
PC1 (84.5%)
Fungivores
PC1 (83.1%)
Herbivores
PC1 (90.4%)
r^2=0.02
r^2=0.04
r^2=0.02
e2
e3
e4
-0.75
-0.65
(B)
Climate
PC1 (66.5%)
-0.13
-0.17
0.52
-0.20
e1
r^2=0.27
-0.26
Ecosystem type
PC1 (29.5%)
0.27
e2
e3
r^2=0.05
r^2=0.07
Resource
PC1 (37.3%)
Soil environment
PC1 (56.8%)
Bacterivores
PC1 (80.8%)
Fungivores
PC1 (82.8%)
Herbivores
PC1 (85.2%)
r^2=0.07
r^2=0.03
r^2=0.07
e4
e5
e6
-0.58
-0.46
-0.44
(C)
-0.13
Climate
PC1 (70.6%)
0.13
0.21
-0.26
0.25
e1
r^2=0.07
Ecosystem type
PC1 (31.4%)
e2
-0.12
e3
r^2=0.05
r^2=0.09
Resource
PC1 (36.2%)
Soil environment
PC1 (55.8%)
0.22
-0.23
Bacterivores
PC1 (83.6%)
Fungivores
PC1 (85.0%)
Herbivores
PC1 (84.8%)
r^2=0.06
r^2=0.06
r^2=0.00
e4
e5
e6
-0.46
-0.48
-0.55
